# Incorporation of recent waking-life experiences in dreams correlates with frontal theta activity in REM sleep

**DOI:** 10.1093/scan/nsy041

**Published:** 2018-06-04

**Authors:** Jean-Baptiste Eichenlaub, Elaine van Rijn, M Gareth Gaskell, Penelope A Lewis, Emmanuel Maby, Josie E Malinowski, Matthew P Walker, Frederic Boy, Mark Blagrove

**Affiliations:** 1 Department of Psychology, Swansea University, Swansea, UK; 2 Department of Psychology, University of York, York, UK; 3 School of Psychological Science, Manchester University, Manchester, UK; 4 Lyon Neuroscience Research Center, INSERM, CNRS, University of Lyon, Lyon, France; 5 Department of Psychology, University of Bedfordshire, Luton, UK; 6 Sleep and Neuroimaging Laboratory, University of California, Berkeley, CA, USA

**Keywords:** frontal theta, dreaming, day-residue effect, REM sleep, memory

## Abstract

Rapid eye movement (REM) sleep and its main oscillatory feature, frontal theta, have been related to the processing of recent emotional memories. As memories constitute much of the source material for our dreams, we explored the link between REM frontal theta and the memory sources of dreaming, so as to elucidate the brain activities behind the formation of dream content. Twenty participants were woken for dream reports in REM and slow wave sleep (SWS) while monitored using electroencephalography. Eighteen participants reported at least one REM dream and 14 at least one SWS dream, and they, and independent judges, subsequently compared their dream reports with log records of their previous daily experiences. The number of references to recent waking-life experiences in REM dreams was positively correlated with frontal theta activity in the REM sleep period. No such correlation was observed for older memories, nor for SWS dreams. The emotional intensity of recent waking-life experiences incorporated into dreams was higher than the emotional intensity of experiences that were not incorporated. These results suggest that the formation of wakefulness-related dream content is associated with REM theta activity, and accords with theories that dreaming reflects emotional memory processing taking place in REM sleep.

## Introduction

Rapid eye movement (REM) sleep and its main oscillatory feature, frontal theta, play a critical role in the processing of recent and emotional memories (for reviews, see Walker, 2009; Walker and van der Helm, 2009; Genzel *et al.*, 2015; Hutchison and Rathore, 2015). Although recent studies indicate that slow wave sleep (SWS) could play a complementary role in this processing (Cairney *et al.*, 2015; Payne *et al.*, 2015), compelling evidence supports the offline benefit of REM sleep for the processing of emotional memories (Wagner *et al.*, 2001; Lara-Carrasco *et al.*, 2009; Gujar *et al.*, 2011; van der Helm *et al.*, 2011; Baran *et al.*, 2012; Payne *et al.*, 2012; Groch *et al.*, 2013, 2015; Spoormaker *et al.*, 2014), and suggests frontal theta activity as a brain mechanism underlying such processing (Nishida *et al.*, 2009; Prehn-Kristensen *et al.*, 2013; Cowdin *et al.*, 2014; Durrant *et al.*, 2015; Seeley *et al.*, 2016; Marquis *et al.*, 2017).

Memories provide one of the core constituents of our dreams. Although waking experiences are not faithfully replayed in dreams (Fosse *et al.*, 2003), dream content arises from waking-life experiences (Stickgold *et al.*, 2001; Nielsen and Stenstrom, 2005; Eichenlaub *et al.*, 2017). The notion that dreaming is connected with experiences of the immediately preceding days was described by Freud using the term ‘day-residues’ (Freud, 1953/1900), and, more recently, numerous studies have refined this phenomenon (Nielsen and Powell, 1989; Powell *et al.*, 1995; Nielsen *et al.*, 2004; Schredl, 2006; Blagrove *et al.*, 2011a,b; van Rijn *et al.*, 2015, 2018; Vallat *et al.*, 2017a). In addition, the nature of the daily experiences incorporated into dreams has been explored, highlighting that emotional and personally significant experiences are preferentially incorporated (Cartwright *et al.*, 1998b, 2006; Schredl, 2006; Propper *et al.*, 2007; Malinowski and Horton, 2014).

This study aimed to explore the link between REM frontal theta activity and the references to recent waking-life experiences in REM dreams, given that ∼80% of awakenings from REM sleep are followed by a dream report (Nielsen, 2000). Following Schredl’s (2006) finding that the majority of waking-life references in dreams are from the previous 2 days, recent experiences were defined as those occurring on the 2 days before the dream, while older memories were defined as from the 8 days before this, all recorded using a 10 day diary. Specifically, we hypothesized that frontal theta power in REM sleep would be positively correlated with number of recent experiences incorporated into REM dreams. By contrast, older experiences and memories, hypothesized to have already been processed in the preceding nights, should no longer be mediated by such oscillations. The association between REM theta activity and wakefulness-related dream content could thus decrease or even disappear for older memories. To test our hypothesis, we explored the correlation between frontal theta power and the number of recent *vs* older wakefulness-related dream incorporations using all the REM dreams collected per participant. In addition, a separate analysis was conducted using the final (i.e. latest) REM dream of each participant, following the view of a sequential processing of memories across the night, as a result of the successive cycles of SWS and REM sleep (Giuditta *et al.*, 1995; Ambrosini and Giuditta, 2001; Walker and Stickgold, 2010; Giuditta, 2014), and accordingly that REM-sleep memory processing would only emerge fully after earlier sleep cycles had been completed.

Since it has been reported that waking-life experiences that are incorporated into dreams are more emotionally intense than those that are not incorporated (Schredl, 2006; Malinowski and Horton, 2014), we also hypothesized a difference in the emotional intensity of incorporated *vs* non-incorporated recent experiences. Our design extends the previous work in this area, in that Malinowski and Horton (2014) assessed emotional intensity but not valence of daily experiences, and these authors and Schredl (2006) had dreams recorded at home, which can be from any sleep-stage. This study assessed both emotional intensity and valence of waking-life experiences, and dream reports were collected after experimental awakenings in REM sleep and SWS.

## Materials and methods

### Participants

Twenty healthy volunteers (10 males, 10 females; mean age = 21.10, s.d. = 3.23, Supplementary Table S1), all students at Swansea University and without sleep disorders or neurological/psychiatric history, participated in the study.

Participants were native English speakers and were frequent dream recallers (i.e. recalling dreams 5–7 times/week). Participants self-reported sleeping a minimum of 7 h per night, not taking recreational drugs and not having an excessive alcohol intake (defined as intake >6 units of alcohol per night or >21 units per week). They were required to abstain from alcohol and drugs throughout the course of the study and were paid for their participation. Ethical approval for the study was obtained from the Research Ethics Committee of the School of Human and Health Sciences, Swansea University, and all participants gave written informed consent to take part.

This study focused on the link between REM frontal theta power and the references to recent experiences in REM dreams, as well as the emotional intensity of these incorporated experiences. The dream content data from the same sleep laboratory participants were used for a study on the dream-lag effect, for which there were three further groups/conditions for home dream collection; that study is presented elsewhere (van Rijn *et al.*, 2015) and these sleep laboratory dreams did not evidence the dream-lag effect.

### Experimental design

**Fig. 1.**
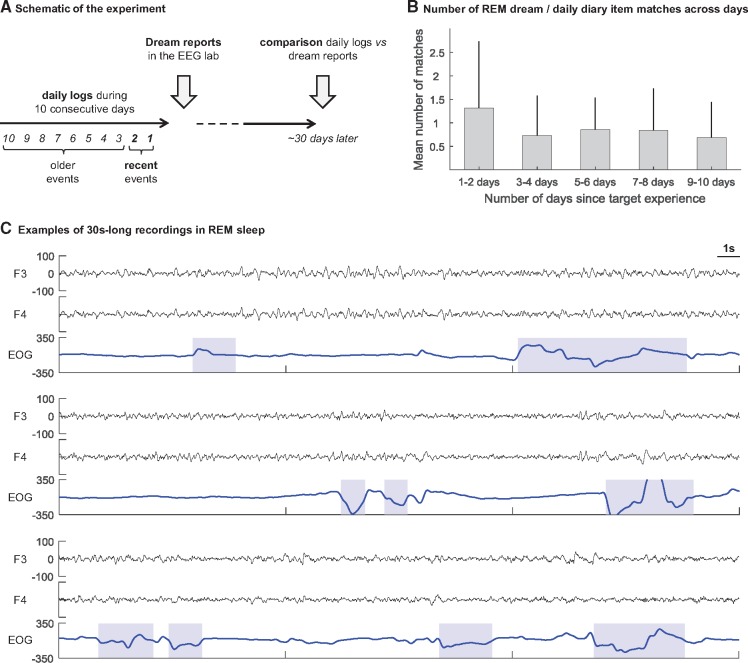
(A) Schematic of the experiment. For 10 consecutive days participants were asked to complete a daily log. On the night of the 10th day, they slept in the sleep laboratory and were monitored using polysomnographic recordings. They were awoken after 10 min of REM or SWS sleep, and were asked to report their dreams. Approximately 4 weeks later they were asked to identify if parts of the dream reports collected in the sleep laboratory matched parts of the 10 daily logs (see Methods). (B) Mean number of waking-life experiences across the 10 daily log days identified by the participants as being incorporated in REM dreams. Per participant, the mean number of incorporations of recent (days 1/2) and older (days 3/4, 5/6, 7/8 and 9/10) waking experiences in REM dreams was calculated (for participants who had only one REM dreams, this unique value was used). The mean across participants was then calculated and is displayed in Figure 1B. Error bars represent s.d. Daily log number refers to the number of days between completing the log and going to the laboratory (e.g. items from daily log 7 occurred 7 days before the night in the laboratory). (C) Examples of 30 s-long recordings (F3, F4 and bipolar EOG) in REM sleep prior to an awakening with a dream report. Three examples from three different participants are displayed. In the dream reports following these REM sleep periods, participants identified 4, 2 and 0 recent incorporations, respectively (from top to bottom panels). EOG are from bipolar montage and the hatched areas characterize EOG events as automatically detected (see Methods).

**Fig. 2.**
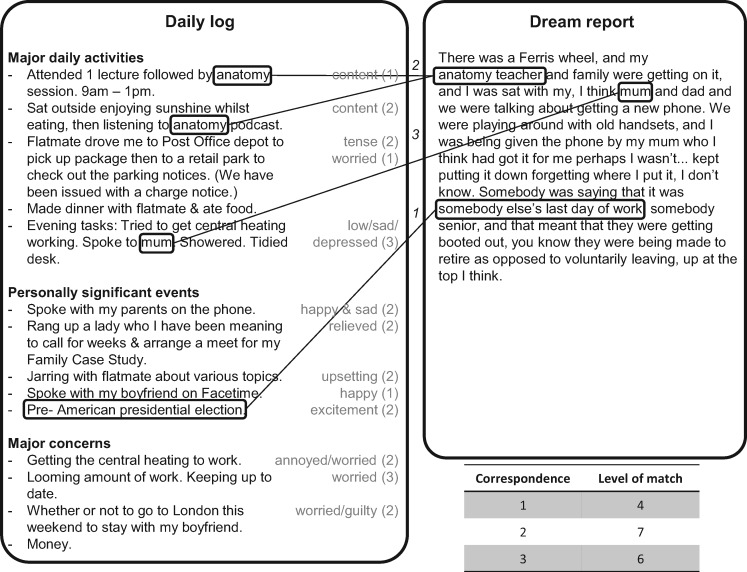
Example of incorporations of daily life experiences into dreams. A daily log is displayed on the left side and a dream report on the right side. In this example, the participant identified three correspondences scored 4, 7 and 6, respectively (on a scale from 0 [none] to 8 [extremely strong] assessing the extent of correspondence between the part of the daily log and the part of the dream report).

The time-course of the experiment is presented in Figure 1A. Participants kept a daily log (Fosse *et al.*, 2003) for 10 consecutive days before sleeping in the laboratory. They were asked to report each evening on the daily log their ‘major daily activities’ (activities that took up most of each participant’s time during the day; e.g. going to work or university, meals, shopping), ‘personally significant events’ (important daily events that may or may not have taken up much time; e.g. emotional events) and ‘major concerns’ (concerns or thoughts that participants had on their mind during the day that may not have taken up much time but were still considered important to them; e.g. money problems, exam stress). Up to five items could be recorded in each category. For each item reported, participants were also instructed to state any accompanying emotion (e.g. anger, anxiety/fear, sadness, shame, joy/elation, love/erotic and surprise) and to rate the intensity of the emotion on a scale, from 1 (low) to 3 (high). An example is displayed in Figure 2.

On the evening of the 10th daily log day participants slept in the sleep laboratory (Supplementary Table S2). During that night, awakenings were conducted in SWS and REM sleep. Awakenings occurred following 10 min of sleep in each sleep-stage. Two SWS awakenings were scheduled during the first and second SWS periods, while REM awakenings were scheduled from the third sleep cycle onwards. If participants recalled a dream from either or both SWS awakenings, they were woken 10 min into every following REM period, until morning. If they failed to recall a dream from the first two SWS awakenings, another SWS awakening was scheduled during the next SWS period. After three SWS awakenings, only REM awakenings followed, regardless of participants’ dream recall from SWS.

Online scoring followed the AASM Manual for the Scoring of Sleep (Iber *et al.*, 2007) and was subsequently confirmed offline. Participants were woken by a buzzer, and interviews were conducted via an intercom. After turning off the buzzer, participants heard the verbal prompt ‘What was going through your mind immediately before you were woken up?’ from a digital voice recorder, and dream reports were recorded using another recorder. To prompt the participants, they were next asked with a recorded message: ‘Can you remember anything else?’. After giving their dream report through the intercom, they were invited to go back to sleep. A researcher blind to the experiment transcribed each dream report.

A habituation night was not included in the design because this would have acted as an event that might be dreamt of during the subsequent experimental night. It is acknowledged that there may thus have been first-night effect physiological changes to sleep structure due to not having a habituation night.

### Correspondence identification task

Approximately 3 weeks after the laboratory night, participants were provided with the materials to perform the correspondence identification task. Participants received their daily logs and dream reports on A3 pages, 29.7 cm (high) × 42.0 cm (wide), each sheet with a daily log on the left side and a dream report on the right, so as to allow comparison of every daily log with every dream report. Each daily log and dream report sheet was assigned a random identifying number and the order of the sheets was randomized. Participants were instructed to compare each of their 10 daily logs with each of their dream report transcripts to identify similarities or correspondences between the log items and dream reports, such as of the characters, objects, actions, locations or themes. If they identified a correspondence, they were instructed to draw boxes around the matched words or sentences in the daily log and around the matched words or sentences in the dream report, and then to rate the level of correspondence between the two parts using a scale from 0 (none) to 8 (extremely strong; Figure 2). Zero, one or more than one correspondence(s) could be identified for each A3 sheet. Examples of sheets and instructions on how to perform the task were provided.

### Behavioral data

The number of daily log items identified by the participants as being incorporated into their dream reports, with scores of 4 or higher, was counted for each daily log and dream report combination. The threshold of 4 was used so as to ensure that participants had confidence in identifying each memory source item used for the analysis. All dream reports were included in the analysis, with no minimum length requirement, as several of the shortest reports were found to exhibit correspondences; furthermore, dream length was controlled for in partial correlation analyses.

Recent waking-life experiences were defined as items from the day of going to the laboratory and the day before that (i.e. from daily logs 1–2, respectively). Older waking-life experiences were defined as items from daily logs 3 to 10 and were analyzed in pairs as respectively daily logs 3–4, 5–6, 7–8 and 9–10. Within each pair of days, daily experiences that occurred on both days and identified as being incorporated into a dream were counted only once, to avoid an overestimation of the frequency of incorporation into dreams.

### Independent judges

Two independent judges conducted blind ratings of the correspondences between dream content and the daily logs of days 1 and 2, thus identifying incorporation of recent waking-life experiences. Ratings were conducted by the judges for these 2 days only because the hypothesized effect was restricted to these days. The judges were asked to identify any elements in the dream reports that matched items in the daily logs, using the same materials, method and instructions as followed by the participants.

The same method of scoring, collation and analysis for the combinations of daily log and dream report was followed for the judge ratings as for the participant ratings. For each daily log and dream report sheet the number of incorporations was computed as the average of the two judges’ scores.

### EEG recordings and spectral analysis

In the laboratory, electroencephalography (EEG), electrooculography (EOG) and electromyography (EMG) were continuously recorded using a Trackit 18/8 system (Lifelines Ltd, UK, sampling rate: 200 Hz). EEG electrodes were placed according to the standard 10–20 system at F3, F4, C3, C4, M1 and M2. EOG electrodes were applied above the right outer canthus and below the left outer canthus, and EMG electrodes on the chin muscles. The common reference electrode was placed on top of the head at CPz position, and the ground electrode on the forehead.

EEG analysis was performed using Fieldtrip (Oostenveld *et al.*, 2011) and MATLAB (The MathWorks, Inc.). Frontal EEG channels (F3 and F4) were filtered between 1 and 25 Hz (high- and low-pass filters, respectively; bidirectional Butterworth, 4th order) and quantitative EEG analysis was performed on the last 3 min of sleep preceding awakenings. EEG traces were visually inspected and awakenings with overall excessive artifacts were excluded.

Each awakening was first epoched in 4 s-long windows with 50% overlap (resulting in 89 4 s-long epochs per awakening) and 4 s-long epochs with absolute signal amplitude exceeding 70 µV in REM sleep and 200 µv in SWS were automatically excluded. Visual inspection was then performed to ensure that all windows exhibiting artifacts were properly excluded. In addition, in REM sleep, only accepted 4 s EEG epochs that were also free of REMs (that is, tonic REM sleep, not overlapping with REM events) were retained, so as to exclude any potential effect of eye movements in our results. REM events were detected in an automatic fashion as described later.

Power spectra were computed by a fast Fourier transform (‘mtmfft’ method in Fieldtrip, demeaning, hanning tapering). The frequency resolution was set at 0.25 Hz, from 1 to 25 Hz. The spectral power density was averaged across accepted 4 s-long epochs and then in the theta band (4–7 Hz) to test our hypothesis. Non-hypothesized spectral power density in the delta (1–3 Hz), alpha (9–13 Hz) and beta (16–25 Hz) bands were also assessed as control wave bands for REM sleep, and theta, delta and sigma (12–16 Hz) for SWS.

### REM detection in REM sleep

Eye movement periods (also called REM events) within the last 3 min of sleep preceding REM awakenings were automatically detected through EOG recordings. EOG channels were filtered between 0.1 and 3 Hz (high- and low-pass filters, respectively; bidirectional Butterworth, 4th order) before being rereferenced to each other (bipolar montage). A detection threshold was then applied (threshold = mean + 2 s.d.; mean and s.d. were estimated after having removed extreme values, i.e. |values| > 400 µV). Zero-crossings immediately before and after periods crossing the threshold were localized and used for further analysis.

A REM event was defined between two consecutive zero-crossings as an event meeting the following conditions: (i) event duration <4 s and (ii) a maximal approximate derivative (from zero-crossing to first sample crossing detection threshold and/or from last sample crossing detection threshold to next zero-crossing; using diff function in MATLAB) of at least 0.2 µV.ms^−1^. All REM events were verified visually and the number and duration of REM events were computed for each awakening.

### Statistical analysis

MATLAB (The MathWorks, Inc.) and SPSS (IBM, USA) were used to perform the statistical analyses.

#### Correlation analyses

The correlations between frontal theta activity and wakefulness-related dream content were tested using Spearman rank-order correlation coefficient and the significance was set at *P* < 0.05 (two-tailed).

For the all REM (and SWS) dreams analyses, where the number of repetitions per participant could vary (from 1 to 4 for REM, and 1 to 2 for SWS), the weighted correlation coefficient as described in Bland and Altman (1995) was computed (using the participant-specific averages for each variable and the number of observations per participant as weights). The significance of this weighted correlation coefficient was determined using a Spearman statistical table (Ramsey, 1989) with *n* = number of participants and df = *n*−2.

To check that time since sleep onset (i.e. sleep duration from the first epoch scored as N2 to the time of instrumental awakening) and length of dream reports (i.e. number of words in the dream report, using the method of Antrobus, 1983) were not confounds in the theta correlations, these parameters were partialled out of the correlation coefficients.

Non-hypothesized relationships between power in other frequency bands and number of incorporations of waking-life memories into REM and SWS dreams were assessed using the same procedure.

#### Emotion of memories incorporated vs not incorporated into dreams

The emotional intensity of daily log items that were incorporated into dream content were compared with the intensity of non-incorporated items, as a function of valence and sleep-stage, using mixed model analysis (two-tailed, *P* < 0.05). To be included in the analysis a participant had to have both incorporated and non-incorporated daily log item(s) for at least one of the four combinations of valence (positive *vs* negative) and sleep-stage (REM *vs* SWS dreams).

## Results

### Sleep measures

One participant failed to fall asleep during the night in the sleep laboratory and one participant did not complete the correspondence task at the end of the experiment, 18 participants were thus considered for further analysis.

In total, 93 awakenings were conducted (mean number of awakenings per participant = 5.2, s.d. = 1.8). Of the 93 awakenings, 48 were conducted in REM (51.6% of the awakenings), 39 in SWS (41.9%) and 6 in a mixed state (e.g. a mix of REM and N2, 6.5%). On average, the REM awakenings occurred 347.9 min (s.d. = 123.6) after sleep onset, while SWS awakenings occurred 110.0 min (s.d. = 92.7) after sleep onset.

### Dream measures

Of the 48 REM awakenings, 46 were followed by a dream report (95.8% of the REM awakenings, mean number of REM dreams per participant = 2.6, range = from 1 to 4 REM dreams; *n* = 18 participants). The REM dream reports contained on average 83.1 words per dream (s.d. = 93.6, min = 3, max = 452). The participants identified on average 1.31 (s.d. = 1.42) recent experiences from days 1 to 2 in REM dreams, and 0.73 (0.86), 0.86 (0.68), 0.84 (0.89) and 0.69 (0.75) older experiences from days 3 to 4, 5 to 6, 7 to 8 and 9 to 10, respectively (Figure 1B).

Of the 39 SWS awakenings, 19 were followed by a dream report (48.7% of the SWS awakenings). Nine participants had one SWS awakening followed by a dream report, and five participants had two SWS awakenings each followed by a dream report. The SWS dream reports contained on average 25.9 words per dream (s.d. = 26.6, min = 1, max = 104). The participants identified on average 1.07 (s.d. = 1.31) recent experiences from days 1 to 2 in SWS dreams, and 0.93 (1.16), 0.79 (0.83), 0.64 (0.99) and 0.93 (1.02) older experiences from days 3 to 4, 5 to 6, 7 to 8 and 9 to 10, respectively.

For the ratings by independent judges of the correspondences between REM dream content and the daily logs from days 1 to 2, a mean of 1.02 recent incorporations (s.d. = 0.89) were identified per participant. Across the 46 REM dreams, the unstandardized Cronbach’s alpha between scores of participants and the judges was 0.78 (considered as acceptable).

### EEG recordings and power spectrum

Two REM awakenings (both followed by a dream report) were excluded from the power analyses due to poor quality EEG recordings/excessive artifact components in the 3 min of sleep preceding the awakening. The EEG analyses were thus performed on the remaining awakenings (46 REM awakenings including 44 followed by a dream report; 39 SWS awakenings including 19 with a dream report). Examples of 30 s-recordings are displayed in Figure 1C.

**Fig. 3.**
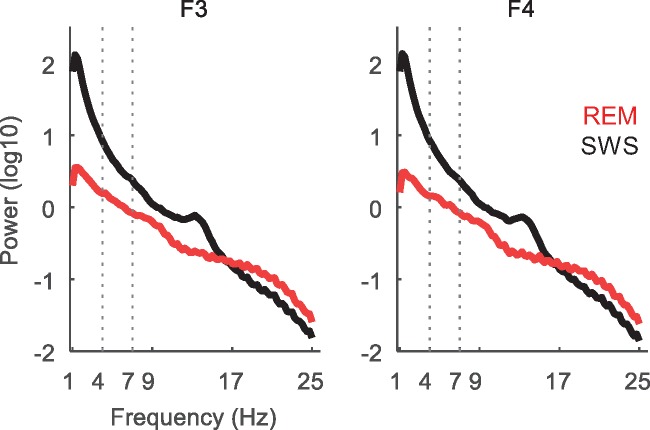
Averaged power spectrum in REM and SWS sleep. Per electrode (F3 and F4), power spectrum of the 3 min of sleep preceding each awakening was computed (see Methods) and log-transformed (base 10) before being averaged across awakenings for REM and SWS separately.

Based on the amplitude threshold and visual inspection, the mean percentage of 4 s-long windows excluded was, respectively, 5.3% (s.d. = 9.7) in REM periods and 1.0% (s.d. = 1.7) in SWS periods. The mean number of EOG events detected in REM periods was 18.3 (s.d. = 6.5; averaged duration within the 3 min = 31.0 s, s.d. = 9.7). Accordingly, the final set of 4 s-long windows accepted in tonic (without REMs) REM periods was defined as the intersection of the 4 s-long windows passing amplitude threshold and without overlapping REMs, resulting in a mean percentage of accepted windows of 62.3% (s.d. = 13.5) in tonic REM periods. The averaged EEG power spectra in (tonic) REM and SWS at F3 and F4 are displayed in Figure 3.

### Correlations between REM frontal theta and number of waking-life memories in REM dreams

**Fig. 4.**
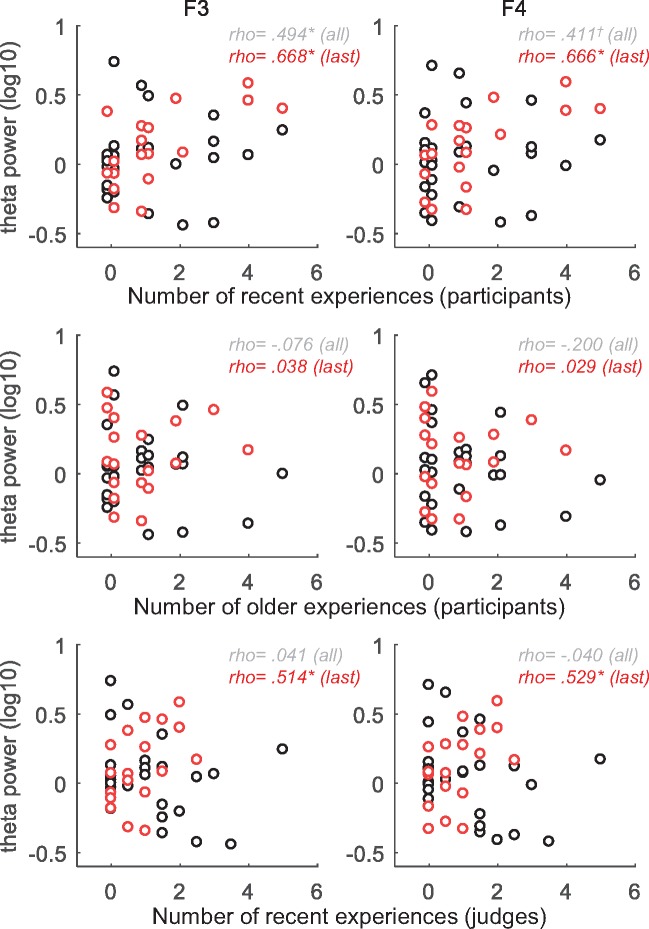
Relationship between the number of incorporations of daily experiences in REM dreams and frontal theta activity in REM sleep. The two first panels display the relationship between the frontal theta power in REM sleep and the number of recent (from days 1/2, top panel) and older (from days 7/8, middle panel) experiences identified as being incorporated in REM dreams by the participants. The last panel displays the relationship when judges independently identified recent incorporations in REM dreams (from days 1/2, bottom panel). Higher frontal theta activity in REM sleep was associated with higher number of references to recent waking experiences in REM dreams as identified by the participants for the all REM (weighted correlation coefficient, in gray) and last/only REM (in red) samples (*P < 0.05, † *P* < 0.10; two-tailed) or by independent judges for the last/only REM samples (*P < 0.05, two-tailed). The sample of the final or only REM dream of each participant is highlighted in red. The data points for number of incorporations 0, 1 and 2 for participants’ scores (top and middle panels) were slightly spaced horizontally for display purposes.

As hypothesized, for the sample of all REM dreams (44 awakenings from 18 participants), the correlation analysis showed that the number of recent waking-life items (from days 1 to 2) incorporated into dream reports was positively correlated with frontal REM theta power (Figure 4, upper panel; F3: weighted Spearman rho = 0.494, *P* < 0.05; F4: weighted rho = 0.411, *P* < 0.10).

No significant correlation was observed between theta power and number of older waking-life log items for either F3 (Figure 4, middle panel; weighted rho = 0.176, 0.179, −0.076 and −0.006, for respectively days 3–4, 5–6, 7–8, 9–10; all *P*-values > 0.20) or F4 (weighted rho = 0.113, 0.185, −0.200, −0.105, for respectively days 3–4, 5–6, 7–8, 9–10; all *P*-values > 0.20). As a further control, correlations between number of incorporations of recent daily log experiences and delta, alpha and beta power in the last 3 min of the REM period were computed. The latter correlations were not significant (delta: F3, weighted rho = 0.441, *P* > 0.05; F4, weighted rho = 0.386, *P* > 0.10; alpha: F3, weighted rho = 0.161, *P* > 0.50; F4, weighted rho = 0.152, *P* > 0.50; beta: F3, weighted rho = 0.162, *P* > 0.50; F4, weighted rho = 0.266 *P* > 0.20).

Partial correlations were computed to adjust for sleep duration preceding the REM awakening, and also to adjust for dream report length. After adjusting for sleep duration, partial weighted correlations (with recent waking-life log items) were rho = 0.507 (*P* < 0.05) at F3 and rho = 0.430 (*P* < 0.10) at F4, while weighted partial correlations were rho = 0.481 (*P* < 0.05) at F3 and rho = 0.390 (*P* < 0.20) at F4 after adjusting for dream report length.

We also analyzed the sample of the last REM dream of each participant (18 awakenings, in red in Figure 4), following the view of a sequential processing of memories across the night, as a result of the successive cycles of SWS and REM sleep (e.g. Giuditta *et al.*, 1995). On average, 1.33 recent incorporations (s.d. = 1.53) were identified per dream, and a significant correlation between the number of recent waking-life log items incorporated and theta power was observed at F3 (rho = 0.668, *P* < 0.005) and F4 (rho = 0.666, *P* < 0.005). After adjusting for sleep duration, partial correlations were rho = 0.667 (*P* < 0.005) at F3 and rho = 0.666 (*P* < 0.005) at F4, while partial correlations were rho = 0.659 (*P* < 0.005) at F3 and rho = 0.667 (*P* < 0.005) at F4 after adjusting for dream report length in words.

### Correlations using external judges

For the sample of all REM dreams, there was no significant correlation between theta and number of recent incorporations identified by judges at F3 (weighted rho = 0.041, *P* > 0.50) or F4 (weighted rho = −0.040, *P* > 0.50).

As was done using the participants’ scores, the sample of the last REM dream of each participant was also explored. On average, 0.86 recent incorporations (s.d. = 0.78) were identified by judges per dream, and a significant correlation between theta and number of incorporations was observed at F3 (rho = 0.514, *P* < 0.05) and F4 (rho = 0.529, *P* < 0.05). So as to investigate whether this difference in comparison to the all REM dreams analysis was related to the length of the dream reports that were included in the analyses, the mean lengths of the two samples of reports were compared. The last or only dream of participants were found to be longer than the earlier dreams of participants [early REM dream reports (*n* = 26) mean = 58.7 words, s.d. = 54.4; last or only REM dream reports (*n* = 18) mean = 113.7 words, s.d. = 128.2] but this difference was not significant [mixed model analysis, *F*(1, 24.9) = 3.46, *P <* 0.10].

### No correlations between EEG power and incorporations in SWS dreams

As predicted, no significant correlation was observed between number of recent incorporations in SWS dreams and SWS theta power for either F3 (weighted rho = 0.219, *P* > 0.20) or F4 (weighted rho = 0.306, *P* > 0.20) analyzing all SWS dreams, or analyzing first/only SWS dream for either F3 (rho = 0.066, *P* > 0.50) or F4 (rho = 0.296, *P* > 0.20). As the main oscillatory feature in SWS is delta activity, these correlations were rerun using delta power. No significant correlation was observed between recent incorporations and SWS delta power for either F3 (weighted rho = 0.289, *P* > 0.20) or F4 (weighted rho = 0.249, *P* > 0.20) analyzing all SWS dreams, or analyzing first/only SWS dream for either F3 (rho = 0.258, *P* > 0.20) or F4 (rho = 0.169, *P* > 0.50). Similarly, no significant correlation was observed between recent incorporations and SWS sigma power for either F3 (rho = 0.093, *P* > 0.50) or F4 (rho = 0.211, *P* > 0.20) analyzing all SWS dreams, or analyzing first/only SWS dream for either F3 (rho = 0.101, *P* > 0.50) or F4 (rho = 0.261, *P* > 0.20).

### Correlations at central channels

Although this study was focused on the relationship between REM frontal theta and the number of recent waking-life experiences in REM dreams, we explored this relationship at central channels as a further control. The correlation at central channels was computed following the same analysis as for frontal channels after having visually excluded EEG traces with overall excessive artifacts. For the sample of all REM dreams, the correlation analysis showed that the number of recent waking-life items (from days 1 to 2) incorporated into dream reports was not significantly correlated with theta power at C3 (36 awakenings from 16 participants; weighted rho = 0.396, *P* > 0.10) or C4 (29 awakenings from 12 participants; weighted rho = 0.429, *P* > 0.10). A positive correlation was observed for the sample of last REM dreams at C3 (15 participants; rho = 0.564, *P* < 0.05) and C4 (11 participants; rho = 0.602, *P* < 0.05). However, using judges’ scores, no significant correlation was observed using all REM dreams (weighted rho = −0.078, *P* > 0.50 at C3 and weighted rho = −0.070, *P* > 0.50 at C4) or last REM dream (rho = 0.315, *P* > 0.20 at C3 and rho = 0.479, *P* > 0.10 at C4). All correlations of central theta with incorporation of older waking-life items were non-significant.

### Emotional intensity of the incorporated *vs* non-incorporated recent items

In total, 372 daily log items from the days 1 to 2 logs were analyzed. The number of occurrences of emotion scores −3, −2, −1, 0, 1, 2 and 3 were 44, 66, 19, 94, 42, 64 and 43, respectively. Items scored from −3 to 0 and from 0 to 3 were classified as negative and positive emotion items, respectively. The means for emotional intensity of incorporated and non-incorporated items are presented in Table 1, as a function of valence of the daily log item and sleep-stage.

**Table 1.** Emotional intensity of the recent experiences as a function of (non-)incorporation into REM *vs* SWS dreams

	Incorporated		Non-incorporated		*n*
	Mean	s.d.	Mean	s.d.	
REM dreams					
Positive emotional items	2.041	0.407	1.739	0.516	12
Negative emotional items	1.961	0.704	1.600	0.764	12
SWS dreams					
Positive emotional items	2.104	0.684	1.505	0.499	8
Negative emotional items	2.167	0.753	1.770	0.581	6

Emotional intensity (mean ± s.d.) of recent daily log items as a function of whether the item was or was not incorporated into a REM or SWS dream, and as a function of valence of the daily log item emotion. Note: Emotions of daily log items were rated −3, −2, −1, 0, +1, +2, +3, and negatively valenced items are represented by absolute value of score. *n* refers to number of participants for each condition.

Fifteen participants had both incorporated and non-incorporated daily log items for at least one of the four combinations of valence (positive *vs* negative) and sleep-stage (REM *vs* SWS dreams) and so were included in the analysis. As many of these participants did not have incorporated and non-incorporated items for all four combinations, the sample sizes shown in Table 1 for each combination are <15. A mixed model analysis using type III tests of fixed effects showed a significant difference between emotional intensity of incorporated and non-incorporated experiences [*F*(1, 28.3) = 11.19, *P* = 0.002] but no interaction between the incorporated/non-incorporated and valence factors [*F*(1, 28.3) = 0.08, *P* > 0.50], nor the incorporated/non-incorporated and sleep-stage factors [*F*(1, 28.3) = 0.44, *P* > 0.50]. The three-way interaction between the incorporated/non-incorporated, valence and sleep-stage factors was also non-significant [*F*(1, 28.3) = 0.29, *P* > 0.50]. Thus, intensity of emotion was associated with incorporation of waking-life experiences into dreams, and this relationship held for positive and negative emotions and for SWS and REM dreams.

## Discussion

The number of references to recent waking-life experiences in REM dreams was found to be positively correlated with frontal theta activity in the last 3 min of the REM sleep period from which the dream was collected. No such correlation was observed for older memories, nor between theta and memory incorporations in SWS dreams, suggesting a REM specific brain correlate of the incorporation of recent waking-life experiences into dreams. The correlations were higher for the last REM dream report of the night. In addition, recent experiences incorporated into REM and SWS dreams had significantly higher emotional intensity than did non-incorporated recent experiences, irrespective of valence.

Although brain activity related to the recall or non-recall of dreams is a field of intense research (Chellappa *et al.*, 2011, 2012; Marzano *et al.*, 2011; Ruby *et al.*, 2013; Eichenlaub *et al.*, 2014a,b; Scarpelli *et al.*, 2017; Vallat *et al.*, 2017b), few studies, to our knowledge, have attempted to link sleeping brain activities with the content of associated dreams. For example, Dresler *et al.* (2011) showed that a predefined motor task performed during REM dreaming was associated with neural activation of the corresponding sensorimotor cortex, Horikawa *et al.* (2013) successfully categorized the dream content of sleepers at N1 sleep onset by applying a machine learning approach on fMRI data, and Siclari *et al.* (2017) linked different components of REM dream content to EEG activity in the corresponding brain areas. These studies identified brain activity during sleep that underlies the content of dreams, providing critical evidence of how dreaming is constructed at the brain level. The findings from this study extend this research by identifying a relationship between REM theta activity and the degree to which dream content is related to recent experiences, with this relationship being present for the whole sample of REM dreams but more so for REM dreams later in the night.

### Dream content and REM-sleep dependent memory consolidation

The literature repeatedly highlights the processing of emotional memories in REM sleep and the relationship of this processing to REM frontal theta oscillations (Wagner *et al.*, 2001; Groch *et al.*, 2013, 2015; Prehn-Kristensen *et al.*, 2013; Cowdin *et al.*, 2014; Durrant *et al.*, 2015; Seeley *et al.*, 2016). Theta power in REM sleep has been correlated with post-sleep recall of recent emotional memories in humans (Nishida *et al.*, 2009), while theta coherence between the amygdala, medial prefrontal cortex and hippocampus in REM sleep has been correlated with overnight bidirectional changes in fear memory in rats (Popa *et al.*, 2010). In this study, the finding that recent experiences incorporated into REM dreams are linked to REM frontal theta, and exhibit higher emotional intensity than the experiences not incorporated, support a framework in which dreaming might be reflective of memory consolidation processes, a framework which has already received support (for reviews, see Stickgold *et al.*, 2001; Payne, 2010; Wamsley, 2014). Our results suggest that the occurrence of recent waking-life experiences in dreams might reflect the ongoing, theta-mediated, consolidation of these new emotional memories in REM sleep. That emotional experiences are more likely to be incorporated into REM and SWS dreams than less emotional experiences accords with theories of the processing of recent emotional memories during sleep, such as Walker and van der Helm’s (2009) Sleep to Remember and Sleep to Forget theory, that sleep causes a reduction in emotional tone while tagged memories are consolidated, as supported by Vallat *et al.*’s (2017a) finding that dream elements have a reduced emotional tone in comparison to their waking-life memory sources. That the last reported REM dream of the night evidenced the relationship with theta more than did earlier REM dreams may be due to a greater complexity of processing once earlier cycles have been completed. Alternatively, this time of night effect may be a function of the greater elaboration, length and complexity of dream-stories in later REM periods (Cipolli and Poli, 1992; Cipolli *et al.*, 2015).

Importantly, this link between frontal theta activity and waking-life experiences in REM dreams was not observed with older memories. This result suggests a transformation across nights of the memories, as proposed by the Sleep to Remember and Sleep to Forget model (Walker, 2009; Walker and van der Helm, 2009). Our results bolster this model by suggesting a transformation across nights of the memory traces processed in REM sleep, that is, while older memories are still present in dreams, these memory traces are no longer related to REM theta activity.

However, the question whether dreaming itself is a functional component of the consolidation of memories is highly debated (Wamsley and Stickgold, 2011; Wamsley, 2014), and this study does not provide conclusive evidence for a memory function of dreaming itself since it is impossible to quantify whether the incorporation of recent waking experiences into REM dreams transformed the memory traces in any meaningful way. Nevertheless, it is important to note that the pioneering work by Cartwright *et al.* (1998a, 2003, 2006) suggests a role of REM dreams in recovery from emotional trauma, while the affective network dysfunction model of Nielsen and Levin (2007) proposes that nightmares reflect failures of a fear memory extinction function of dreaming. These works highlight a memory function of dreaming through the regulation of emotional components of memories, and the current results are compatible with this hypothesis that a function of REM sleep, and concomitant dreaming, is to transform emotional memories.

### Identification of incorporations by external judges

Incorporations of recent experiences identified by judges correlated with frontal theta power for the sample comprising the final REM dream of each participant, while no correlation was observed when all REM dreams were considered. Caution is needed, however, regarding this lack of correlation for judge identified incorporations when early REM dreams were included in analyses. Despite the acceptable reliability between judges and participant scores, the relative shortness in length of early REM dream reports might have resulted in judges having less detail on which to base judgments about dream content than do participants themselves, a point discussed by Sikka *et al.* (2014).

### Limitations of the study

Although there was no significant correlation for SWS dreams, there were also fewer dream reports in that condition compared with REM sleep, raising the possibility that the absence of a correlation could be due to low statistical power. Furthermore, the use of a multiple-night design is recommended for future studies so as to limit the sleep disturbance due to multiple awakenings within a single night as used in this study, affording an across-night balance of both sleep-stages and sleep cycles, and greater representativeness of REM dreams for the different cycles.

Although the rationale for the findings of this study concerned frontal theta, we acknowledge that as we did not have a full array of control regions for EEG, it was not possible to fully explore to what extent the effect was specific to the frontal region. The effect was also found for neighboring central electrodes using the last REM sample, although not significantly so for the all REM sample nor for judge scored incorporations. That the effect might be present for central regions is plausible. By comparing REM sleep awakenings that were followed by a dream report *vs* those not followed by a dream report, Marzano *et al.* (2011) reported differences in theta power between the two conditions that exhibited a topography spreading to central and left temporal areas, although only in frontal areas was the association of theta power with dream recall significant. Accordingly, future studies should use a larger number of electrodes to refine the involvement of frontal and neighboring central and temporal regions in the correlation between the number of recent waking-life experiences incorporated into REM dreams and REM theta power.

## Conclusion

The number of references to recent waking-life experiences in REM dreams was found to be positively correlated with frontal theta activity in the REM sleep period. No such correlation was observed for older memories, nor for SWS dreams. Furthermore, the emotional intensity of recent waking-life experiences incorporated into dreams was higher than the emotional intensity of experiences that were not incorporated. These results accord with theories that dreaming reflects emotional memory processing taking place in REM sleep.

## Funding

Study funded by UK Economic and Social Research Council award ES/I037555/1 Dream content as a measure of memory consolidation across multiple periods of sleep.

## Supplementary data

Supplementary data are available at *SCAN* online.

Conflict of interest. None declared.

## Supplementary Material

Supplementary Data

## References

[nsy041-B1] Ambrosini M.V., Giuditta A. (2001). Learning and sleep: the sequential hypothesis. Sleep Medicine Reviews, 5(6), 477–90. 12531155 10.1053/smrv.2001.0180

[nsy041-B2] Antrobus J. (1983). REM and NREM sleep reports: comparison of word frequencies by cognitive classes. Psychophysiology, 20(5), 562–8. 6635096 10.1111/j.1469-8986.1983.tb03015.x

[nsy041-B3] Baran B., Pace-Schott E.F., Ericson C., Spencer R.M. (2012). Processing of emotional reactivity and emotional memory over sleep. Journal of Neuroscience, 32(3), 1035–42. 22262901 10.1523/JNEUROSCI.2532-11.2012 PMC3548452

[nsy041-B4] Blagrove M., Fouquet N.C., Henley-Einion J.A., et al (2011a). Assessing the dream-lag effect for REM and NREM stage 2 dreams. PLoS One, 6(10), e26708. 22046336 10.1371/journal.pone.0026708 PMC3202556

[nsy041-B5] Blagrove M., Henley-Einion J., Barnett A., Edwards D., Heidi Seage C. (2011b). A replication of the 5-7 day dream-lag effect with comparison of dreams to future events as control for baseline matching. Consciousness and Cognition, 20(2), 384–91. 20739193 10.1016/j.concog.2010.07.006

[nsy041-B6] Bland J.M., Altman D.G. (1995). Calculating correlation coefficients with repeated observations: part 2–correlation between subjects. BMJ, 310(6980), 633. 7703752 10.1136/bmj.310.6980.633 PMC2549010

[nsy041-B7] Cairney S.A., Durrant S.J., Power R., Lewis P.A. (2015). Complementary roles of slow-wave sleep and rapid eye movement sleep in emotional memory consolidation. Cerebral Cortex, 25(6), 1565–75. 24408956 10.1093/cercor/bht349

[nsy041-B8] Cartwright R., Agargun M.Y., Kirkby J., Friedman J.K. (2006). Relation of dreams to waking concerns. Psychiatry Research, 141(3), 261–70. 16497389 10.1016/j.psychres.2005.05.013

[nsy041-B9] Cartwright R., Baehr E., Kirkby J., Pandi-Perumal S.R., Kabat J. (2003). REM sleep reduction, mood regulation and remission in untreated depression. Psychiatry Research, 121(2), 159–67. 14656450 10.1016/s0165-1781(03)00236-1

[nsy041-B10] Cartwright R., Luten A., Young M., Mercer P., Bears M. (1998). Role of REM sleep and dream affect in overnight mood regulation: a study of normal volunteers. Psychiatry Research, 81(1), 1–8. 9829645 10.1016/s0165-1781(98)00089-4

[nsy041-B11] Cartwright R., Young M.A., Mercer P., Bears M. (1998). Role of REM sleep and dream variables in the prediction of remission from depression. Psychiatry Research, 80(3), 249–55. 9796940 10.1016/s0165-1781(98)00071-7

[nsy041-B12] Chellappa S.L., Frey S., Knoblauch V., Cajochen C. (2011). Cortical activation patterns herald successful dream recall after NREM and REM sleep. Biological Psychology, 87(2), 251–6. 21419827 10.1016/j.biopsycho.2011.03.004

[nsy041-B13] Chellappa S.L., Munch M., Knoblauch V., Cajochen C. (2012). Age effects on spectral electroencephalogram activity prior to dream recall. Journal of Sleep Research, 21(3), 247–56. 21851439 10.1111/j.1365-2869.2011.00947.x

[nsy041-B14] Cipolli C., Guazzelli M., Bellucci C., et al (2015). Time-of-night variations in the story-like organization of dream experience developed during rapid eye movement sleep. Journal of Sleep Research, 24(2), 234–40. 25307048 10.1111/jsr.12251

[nsy041-B15] Cipolli C., Poli D. (1992). Story structure in verbal reports of mental sleep experience after awakening in REM sleep. Sleep, 15(2), 133–42. 1579787 10.1093/sleep/15.2.133

[nsy041-B16] Cowdin N., Kobayashi I., Mellman T.A. (2014). Theta frequency activity during rapid eye movement (REM) sleep is greater in people with resilience versus PTSD. Experimental Brain Research, 232(5), 1479–85. 24531640 10.1007/s00221-014-3857-5 PMC4449337

[nsy041-B17] Dresler M., Koch S.P., Wehrle R., et al (2011). Dreamed movement elicits activation in the sensorimotor cortex. Current Biology, 21(21), 1833–7. 22036177 10.1016/j.cub.2011.09.029

[nsy041-B18] Durrant S.J., Cairney S.A., McDermott C., Lewis P.A. (2015). Schema-conformant memories are preferentially consolidated during REM sleep. Neurobiology of Learning and Memory, 122, 41–50. 25754499 10.1016/j.nlm.2015.02.011

[nsy041-B19] Eichenlaub J.B., Bertrand O., Morlet D., Ruby P. (2014a). Brain reactivity differentiates subjects with high and low dream recall frequencies during both sleep and wakefulness. Cerebral Cortex, 24(5), 1206–15. 23283685 10.1093/cercor/bhs388

[nsy041-B20] Eichenlaub J.B., Cash S.S., Blagrove M. (2017). Daily life experiences in dreams and sleep-dependent memory consolidation In: Axmacher N., Rasch B., editors. Cognitive Neuroscience of Memory Consolidation, Switzerland: Springer Cham, pp. 161–72.

[nsy041-B21] Eichenlaub J.-B., Nicolas A., Daltrozzo J., Redouté J., Costes N., Ruby P. (2014b). Resting brain activity varies with dream recall frequency between subjects. Neuropsychopharmacology, 39(7), 1594–602. 24549103 10.1038/npp.2014.6 PMC4023156

[nsy041-B22] Fosse M.J., Fosse R., Hobson J.A., Stickgold R.J. (2003). Dreaming and episodic memory: a functional dissociation? Journal of Cognitive Neuroscience, 15(1), 1–9. 12590838 10.1162/089892903321107774

[nsy041-B23] Freud S. (1953/1900). The Interpretation of Dreams (Trans. J. Stratchey). London: Hogarth Press (Original Publication date 1900).

[nsy041-B24] Genzel L., Spoormaker V.I., Konrad B.N., Dresler M. (2015). The role of rapid eye movement sleep for amygdala-related memory processing. Neurobiology of Learning and Memory, 122, 110–21. 25638277 10.1016/j.nlm.2015.01.008

[nsy041-B25] Giuditta A. (2014). Sleep memory processing: the sequential hypothesis. Frontiers in Systems Neuroscience, 8, 219. 25565985 10.3389/fnsys.2014.00219 PMC4267175

[nsy041-B26] Giuditta A., Ambrosini M.V., Montagnese P., et al (1995). The sequential hypothesis of the function of sleep. Behavioural Brain Research, 69(1–2), 157–66. 7546307 10.1016/0166-4328(95)00012-i

[nsy041-B27] Groch S., Wilhelm I., Diekelmann S., Born J. (2013). The role of REM sleep in the processing of emotional memories: evidence from behavior and event-related potentials. Neurobiology of Learning and Memory, 99, 1–9. 23123802 10.1016/j.nlm.2012.10.006

[nsy041-B28] Groch S., Zinke K., Wilhelm I., Born J. (2015). Dissociating the contributions of slow-wave sleep and rapid eye movement sleep to emotional item and source memory. Neurobiology of Learning and Memory, 122, 122–30. 25180933 10.1016/j.nlm.2014.08.013

[nsy041-B29] Gujar N., McDonald S.A., Nishida M., Walker M.P. (2011). A role for REM sleep in recalibrating the sensitivity of the human brain to specific emotions. Cerebral Cortex, 21(1), 115–23. 20421251 10.1093/cercor/bhq064 PMC3000566

[nsy041-B30] Horikawa T., Tamaki M., Miyawaki Y., Kamitani Y. (2013). Neural decoding of visual imagery during sleep. Science, 340(6132), 639–42. 23558170 10.1126/science.1234330

[nsy041-B31] Hutchison I.C., Rathore S. (2015). The role of REM sleep theta activity in emotional memory. Frontiers in Psychology, 6, 1439. 26483709 10.3389/fpsyg.2015.01439 PMC4589642

[nsy041-B32] Iber C., Ancoli-Israel S., Chesson A., Quan S.F. (2007). The AASM Manual for the Scoring of Sleep and Associated Events: Rules, Terminology, and Technical Specification. Westchester, IL: American Academy of Sleep Medicine.

[nsy041-B33] Lara-Carrasco J., Nielsen T.A., Solomonova E., Levrier K., Popova A. (2009). Overnight emotional adaptation to negative stimuli is altered by REM sleep deprivation and is correlated with intervening dream emotions. Journal of Sleep Research, 18(2), 178–87. 19645964 10.1111/j.1365-2869.2008.00709.x

[nsy041-B34] Malinowski J.E., Horton C.L. (2014). Evidence for the preferential incorporation of emotional waking-life experiences into dreams. Dreaming, 24(1), 18–31.

[nsy041-B35] Marquis L.P., Paquette T., Blanchette-Carriere C., Dumel G., Nielsen T. (2017). REM sleep theta changes in frequent nightmare recallers. Sleep, 40(9), zsx110. 10.1093/sleep/zsx110 PMC5806577 28651358

[nsy041-B36] Marzano C., Ferrara M., Mauro F., et al (2011). Recalling and forgetting dreams: theta and alpha oscillations during sleep predict subsequent dream recall. Journal of Neuroscience, 31(18), 6674–83. 21543596 10.1523/JNEUROSCI.0412-11.2011 PMC6632857

[nsy041-B37] Nielsen T.A. (2000). A review of mentation in REM and NREM sleep: “covert” REM sleep as a possible reconciliation of two opposing models. Behavioral and Brain Sciences, 23(6), 851–66. discussion 904–1121. 11515145 10.1017/s0140525x0000399x

[nsy041-B38] Nielsen T.A., Kuiken D., Alain G., Stenstrom P., Powell R.A. (2004). Immediate and delayed incorporations of events into dreams: further replication and implications for dream function. Journal of Sleep Research, 13(4), 327–36. 15560767 10.1111/j.1365-2869.2004.00421.x

[nsy041-B39] Nielsen T.A., Levin R. (2007). Nightmares: a new neurocognitive model. Sleep Medicine Reviews, 11(4), 295–310. 17498981 10.1016/j.smrv.2007.03.004

[nsy041-B40] Nielsen T.A., Powell R.A. (1989). The ‘dream-lag’ effect: a 6-day temporal delay in dream content incorporation. Psychiatric Journal of the University of Ottawa: Revue De Psychiatrie De L’Universite D’Ottawa, 14(4), 561–5. 2813638

[nsy041-B41] Nielsen T.A., Stenstrom P. (2005). What are the memory sources of dreaming? Nature, 437(7063), 1286–9. 16251954 10.1038/nature04288

[nsy041-B42] Nishida M., Pearsall J., Buckner R.L., Walker M.P. (2009). REM sleep, prefrontal theta, and the consolidation of human emotional memory. Cerebral Cortex, 19(5), 1158–66. 18832332 10.1093/cercor/bhn155 PMC2665156

[nsy041-B43] Oostenveld R., Fries P., Maris E., Schoffelen J.M. (2011). FieldTrip: open source software for advanced analysis of MEG, EEG, and invasive electrophysiological data. Computational Intelligence and Neuroscience, 2011, 1. 21253357 10.1155/2011/156869 PMC3021840

[nsy041-B44] Payne J.D. (2010). Memory consolidation, the diurnal rhythm of cortisol, and the nature of dreams: a new hypothesis. International Review of Neurobiology, 92, 101–34. 20870065 10.1016/S0074-7742(10)92006-0

[nsy041-B45] Payne J.D., Chambers A.M., Kensinger E.A. (2012). Sleep promotes lasting changes in selective memory for emotional scenes. Frontiers in Integrative Neuroscience, 6, 108. 23181013 10.3389/fnint.2012.00108 PMC3503264

[nsy041-B46] Payne J.D., Kensinger E.A., Wamsley E.J., et al (2015). Napping and the selective consolidation of negative aspects of scenes. Emotion, 15(2), 176–86. 25706830 10.1037/a0038683 PMC5846328

[nsy041-B47] Popa D., Duvarci S., Popescu A.T., Lena C., Pare D. (2010). Coherent amygdalocortical theta promotes fear memory consolidation during paradoxical sleep. Proceedings of the National Academy of Sciences of the United States of America, 107(14), 6516–9. 20332204 10.1073/pnas.0913016107 PMC2851973

[nsy041-B48] Powell R.A., Cheung J.S., Nielsen T.A., Cervenka T.M. (1995). Temporal delays in incorporation of events into dreams. Perceptual and Motor Skills, 81(1), 95–104. 8532489 10.2466/pms.1995.81.1.95

[nsy041-B49] Prehn-Kristensen A., Munz M., Molzow I., Wilhelm I., Wiesner C.D., Baving L. (2013). Sleep promotes consolidation of emotional memory in healthy children but not in children with attention-deficit hyperactivity disorder. PLoS One, 8(5), e65098. 23734235 10.1371/journal.pone.0065098 PMC3667133

[nsy041-B50] Propper R.E., Stickgold R., Keeley R., Christman S.D. (2007). Is television traumatic? Dreams, stress, and media exposure in the aftermath of September 11, 2001. Psychological Science, 18(4), 334–40. 17470259 10.1111/j.1467-9280.2007.01900.x

[nsy041-B51] Ramsey P.H. (1989). Critical values for Spearman’s rank order correlation. Journal of Educational Statistics, 14(3), 245–53.

[nsy041-B52] Ruby P., Blochet C., Eichenlaub J.B., Bertrand O., Morlet D., Bidet-Caulet A. (2013). Alpha reactivity to first names differs in subjects with high and low dream recall frequency. Frontiers in Psychology, 4, 419. 23966960 10.3389/fpsyg.2013.00419 PMC3743036

[nsy041-B53] Scarpelli S., D’Atri A., Mangiaruga A., et al (2017). Predicting dream recall: EEG activation during NREM sleep or shared mechanisms with wakefulness? Brain Topography, 30(5), 629–38. 28434101 10.1007/s10548-017-0563-1

[nsy041-B54] Schredl M. (2006). Factors affecting the continuity between waking and dreaming: emotional intensity and emotional tone of the waking-life event. Sleep and Hypnosis, 8(1), 1–5.

[nsy041-B55] Seeley C.J., Smith C.T., MacDonald K.J., Beninger R.J. (2016). Ventromedial prefrontal theta activity during rapid eye movement sleep is associated with improved decision-making on the Iowa gambling task. Behavioral Neuroscience, 130(3), 271–80. 26820586 10.1037/bne0000123

[nsy041-B56] Siclari F., Baird B., Perogamvros L., et al (2017). The neural correlates of dreaming. Nature Neuroscience, 20(6), 872–8. 28394322 10.1038/nn.4545 PMC5462120

[nsy041-B57] Sikka P., Valli K., Virta T., Revonsuo A. (2014). I know how you felt last night, or do I? Self- and external ratings of emotions in REM sleep dreams. Consciousness and Cognition, 25, 51–66. 24565868 10.1016/j.concog.2014.01.011

[nsy041-B58] Spoormaker V.I., Gvozdanovic G.A., Samann P.G., Czisch M. (2014). Ventromedial prefrontal cortex activity and rapid eye movement sleep are associated with subsequent fear expression in human subjects. Experimental Brain Research, 232(5), 1547–54. 24452776 10.1007/s00221-014-3831-2

[nsy041-B59] Stickgold R., Hobson J.A., Fosse R., Fosse M. (2001). Sleep, learning, and dreams: off-line memory reprocessing. Science, 294(5544), 1052–7. 11691983 10.1126/science.1063530

[nsy041-B60] Vallat R., Chatard B., Blagrove M., Ruby P. (2017a). Characteristics of the memory sources of dreams: a new version of the content-matching paradigm to take mundane and remote memories into account. PLoS One, 12(10), e0185262. 29020066 10.1371/journal.pone.0185262 PMC5636081

[nsy041-B61] Vallat R., Lajnef T., Eichenlaub J.B., et al (2017b). Increased evoked potentials to arousing auditory stimuli during sleep: implication for the understanding of dream recall. Frontiers in Human Neuroscience, 11, 132. 28377708 10.3389/fnhum.2017.00132 PMC5360011

[nsy041-B62] van der Helm E., Yao J., Dutt S., Rao V., Saletin J.M., Walker M.P. (2011). REM sleep depotentiates amygdala activity to previous emotional experiences. Current Biology, 21(23), 2029–32. 22119526 10.1016/j.cub.2011.10.052 PMC3237718

[nsy041-B63] van Rijn E., Eichenlaub J.B., Lewis P.A., et al (2015). The dream-lag effect: selective processing of personally significant events during rapid eye movement sleep, but not during slow wave sleep. Neurobiology of Learning and Memory, 122, 98–109. 25683202 10.1016/j.nlm.2015.01.009

[nsy041-B64] van Rijn E., Reid A.M., Edwards C.L., et al (2018). Daydreams incorporate recent waking life concerns but do not show delayed (‘dream-lag’) incorporations. Consciousness and Cognition, 58, 51–9. 29128282 10.1016/j.concog.2017.10.011

[nsy041-B65] Wagner U., Gais S., Born J. (2001). Emotional memory formation is enhanced across sleep intervals with high amounts of rapid eye movement sleep. Learning & Memory, 8(2), 112–9. 11274257 10.1101/lm.36801 PMC311359

[nsy041-B66] Walker M.P. (2009). The role of sleep in cognition and emotion. Annals of the New York Academy of Sciences, 1156, 168–97. 19338508 10.1111/j.1749-6632.2009.04416.x

[nsy041-B67] Walker M.P., Stickgold R. (2010). Overnight alchemy: sleep-dependent memory evolution. Nature Reviews Neuroscience, 11(3), 218; author reply 218. 10.1038/nrn2762-c1 PMC2891532 20168316

[nsy041-B68] Walker M.P., van der Helm E. (2009). Overnight therapy? The role of sleep in emotional brain processing. Psychological Bulletin, 135(5), 731–48. 19702380 10.1037/a0016570 PMC2890316

[nsy041-B69] Wamsley E.J. (2014). Dreaming and offline memory consolidation. Current Neurology and Neuroscience Reports, 14(3), 433. 24477388 10.1007/s11910-013-0433-5 PMC4704085

[nsy041-B70] Wamsley E.J., Stickgold R. (2011). Memory, sleep and dreaming: experiencing consolidation. Sleep Medicine Clinics, 6(1), 97–108. 21516215 10.1016/j.jsmc.2010.12.008 PMC3079906

