# Supplementary material for: Incorporation of recent waking-life experiences in dreams correlates with frontal theta activity in REM sleep
Source: Soc Cogn Affect Neurosci. 2018 Jun 4;13(6):637–47. doi: 10.1093/scan/nsy041 (PMC6022568; doi:10.1093/scan/nsy041)
Supplement: Supplementary Data [file nsy041_scan-17-454-file006.docx]

# Supplementary Information for

**Incorporation of recent waking-life experiences in dreams correlates with frontal theta activity in REM sleep**

*Jean-Baptiste Eichenlaub, Elaine van Rijn, M. Gareth Gaskell, Penelope A. Lewis, Emmanuel Maby, Josie Malinowski, Matthew P. Walker, Frederic Boy, and Mark Blagrove*

# Supplementary Tables

| **Participants' Information** | | | | | | | | |
| --- | --- | --- | --- | --- | --- | --- | --- | --- |
|  | P# | Sex | Age | Department | Year | UST (min) | *estimated* DRF | *measured* DRF |
|  | **1** | M | 22 | Sports Science | 3 | 570 | 5-7 | 7.0 |
|  | **2** | F | 27 | Nursing | 1 | 510 | 5-7 | 4.2 |
|  | **3** | F | 18 | Medicine | 1 | 540 | 5-7 | 5.6 |
|  | **4** | M | 18 | Psychology | 1 | 600 | 5-7 | 5.6 |
|  | **5** | F | 20 | Medicine | 1 | 420 | 5-7 | 7.0 |
|  | **6** | M | 24 | Law | 4 | 420 | 5-7 | 7.0 |
|  | **7** | F | 18 | Arts & Humanities | 1 | 450 | 5-7 | 5.6 |
|  | **8** | M | 19 | Arts & Humanities | 1 | 540 | 5-7 | 7.0 |
|  | **9** | M | 31 | Psychology | 7 | 480 | 5-7 | 5.6 |
|  | **10** | F | 20 | History | 2 | 540 | 5-7 | 5.6 |
|  | **11** | F | 19 | Psychology | 2 | 540 | 5-7 | 6.3 |
|  | **12** | M | 21 | Criminology | 1 | 420 | 5-7 | 5.6 |
|  | **13** | M | 21 | Engineering | 3 | 480 | 5-7 | 4.2 |
|  | **14** | F | 19 | Psychology | 1 | 510 | 5-7 | 7.0 |
|  | **15** | M | 21 | Psychology | 3 | 420 | 5-7 | 7.0 |
|  | **16** | F | 19 | Business & Eco. | 1 | 480 | 5-7 | 7.0 |
|  | **17** | M | 22 | Maths & German | 3 | 480 | 5-7 | 3.5 |
|  | **18** | F | 19 | Psychology | 2 | 570 | 5-7 | 5.6 |
|  | **19** | M | 22 | Arts & Humanities | 4 | 480 | 5-7 | 6.3 |
|  | **20** | F | 22 | Medicine | 1 | 540 | 5-7 | 7.0 |
| *mean* | |  | 21.1 |  | 2.2 | 499.5 |  | 6.0 |
| *SD* | |  | 3.2 |  | 1.6 | 55.3 |  | 1.1 |

**Supplementary table 1.** Twenty participants (P#) were recruited. Sex: Male (M) or Female (F). Age (years). Department and Year of Study: all the participants were students enrolled at Swansea University in different departments and in different years of study. Usual Sleep Time (UST): self-evaluation of usual sleep duration (in minutes). *Estimated* DRF: retrospective dream recall frequency reported by participants before taking part in the study (during the recruitment processing) and using the following multiple choice question: “Usually, how often do you wake up and are able to remember a dream in detail? *5-7times/week *2-4times/week *~1/week *1-3times/month *Less than once/month *Almost never”. *Measured* DRF: prospective dream recall frequency assessed from a dream log kept during 10 days following the night in the laboratory (both DRFs are expressed in number of times/mornings per week with dream recalled on awakening).

| **Sleep Parameters** | | |
| --- | --- | --- |
| Variables | *mean* | *SD* |
| SPT (min) | 475.6 | 73.0 |
| TST (min) | 359.4 | 70.4 |
| TST (%) | 75.4 | 9.1 |
| NREM N1 (min) | 23.7 | 13.9 |
| NREM N1 (%) | 4.9 | 2.8 |
| NREM N2 (min) | 187.8 | 37.1 |
| NREM N2 (%) | 39.6 | 5.4 |
| NREM N3 (min) | 103.5 | 38.2 |
| NREM N3 (%) | 21.6 | 7.0 |
| REM (min) | 44.4 | 15.3 |
| REM (%) | 9.3 | 3.2 |
| Wake (min) | 90.4 | 52.4 |
| Wake (%) | 18.6 | 9.8 |
| Mvts/ind (min) | 25.8 | 15.2 |
| Mvts/ind (%) | 5.9 | 5.2 |

**Supplementary table 2.** Average time (± SD) spent in the different states of vigilance during the night in the sleep laboratory (in minutes and percentage). Sleep Period Time (SPT): time from the first (N2) to the last epoch of sleep. Total Sleep Time (TST): sum of the 4 sleep-stages (NREM N1, N2, N3 and REM). Movements/Indeterminate (Mvts/ind): time defined as movements and/or indeterminate.
